# Comparison of the Panther Fusion and Allplex assays for the detection of respiratory viruses in clinical samples

**DOI:** 10.1371/journal.pone.0226403

**Published:** 2019-12-27

**Authors:** Lola Folgueira, Noelia Moral, Consuelo Pascual, Rafael Delgado

**Affiliations:** 1 Microbiology Department, Hospital Universitario 12 de Octubre, Madrid, Spain; 2 Biomedical Research Institute imas12, Hospital Universitario 12 de Octubre, Madrid, Spain; 3 Department of Medicine, School of Medicine, Universidad Complutense, Madrid, Spain; Defense Threat Reduction Agency, UNITED STATES

## Abstract

Respiratory viral infections are the most frequent clinical syndrome affecting both children and adults, and early detection is fundamental to avoid infection-related risks and reduce the healthcare costs incurred by unnecessary antibiotic treatments. In this study, performance characteristics of two commercial methods, the Panther Fusion® assay (Hologic Inc., San Diego, CA, USA) were compared to Allplex^™^ respiratory panels (Seegene, Seoul, South Korea) for the detection of influenza A (Flu A), influenza B (Flu B), respiratory syncytial virus (RSV), parainfluenza virus (PIV), human metapneumovirus (hMPV), rhinovirus (RV) and adenovirus (AdV) targets. A total of 865 specimens collected prospectively and retrospectively were included, and discordant results were further examined using another commercial product, R-GENE^™^ respiratory kits (bioMérieux, Marcy l’Etoile, France). There was high agreement between both methods, with 98.6% concordance and a kappa (k) value of 0.9 (95% CI: 0.89–0.92). A specific analysis of both methods for each viral agent demonstrated comparable sensitivity and specificity, both ranging from 0.83 to 1 with good predictive values for the prospective part of the study. Good agreement between both methods was also found for the κ values obtained (ranging from 97.55% to 98.9%), with the lowest for hMPV (k = 0.83, 95% CI: 0.75–0.91) and RV (k = 0.73, 95% CI: 0.65–0.81). Amplification efficiency, measured according to the value of the cycle threshold (C_t_) obtained in each of the amplifications in both tests, was significantly better with Panther Fusion for Flu A, Flu B, hMPV and RV. Regarding discordant results, R-GENE showed higher agreement with Panther Fusion-positive specimens (negative for Allplex; *n* = 28/71, 34.9%) than with Allplex-positive samples (negative for Panther Fusion; *n* = 7/49, 14.3%). In summary, Panther Fusion proved to be a more efficient fully-automated methodology, requiring shorter hands-on and turnaround times than Allplex.

## Introduction

Viruses are the most common etiologic agents of respiratory infections [1], which cause comorbidity and mortality in both children and adults [2–7]. Consequently, early detection of respiratory viruses is fundamental for reducing risks associated with infection and nosocomial transmission, and to avoid inappropriate treatments and time-consuming laboratory testing [8–10].

The implementation of nucleic acid amplification tests (NAATs) has revolutionized the diagnosis of viral respiratory tract infections due to their high sensitivity and specificity, rapid virus identification, and the possibility of detecting pathogens that could not be identified by conventional diagnostic methods [11, 12]. However, many of the commercially available tests have focused on the detection of influenza and respiratory syncytial virus (RSV) and have been used in point-of-care facilities, limiting the number of samples that can be analyzed simultaneously. Therefore, there is a need for rapid, fully automated commercial techniques that allow detection of a broad panel of respiratory viruses, can be adapted to variable sample sizes and would enable the application of diagnostic algorithms [12] to consider parameters such as patient characteristics (immunosuppression, basal respiratory diseases) and seasonality of the viruses. Among the commercial systems currently available, Allplex^™^ (Seegene, Seoul, South Korea) offers simultaneous detection of 18 viral targets, including all respiratory virus of medical importance, and also provides epidemiological information, as it detects influenza A subtypes, RSV and parainfluenza virus types, as well as the identification of 3 of the 4 coronaviruses (229E, NL63 and OC43) that produce respiratory infection in humans. This assay allows the differentiated detection of enterovirus and picornavirus, a singular feature among commercial platforms that perform syndromic diagnosis of viral respiratory infection. These characteristics made it the routine method implemented in our laboratory starting in the 2016–2017 flu season. Allplex applies its own technology (multiple detection temperature, MuDT^^™^^) that allows the simultaneous amplification of multiple targets at the same wavelength without melt curve analysis.

The Panther Fusion® platform [Hologic Inc., San Diego, CA, USA] was recently released to the market, presenting sample-to-result automation as one of its main characteristics. Although it detects a smaller number of viral targets, the reduced response time, random access and possibility of testing samples urgently, are sufficiently attractive arguments to consider its clinical use.

The purpose of this study was to compare these two commercial products (Panther Fusion assay and Allplex respiratory panels) for the detection of influenza A (Flu A), influenza B (Flu B), RSV, parainfluenza virus (PIV), human metapneumovirus (hMPV), rhinovirus (RV) and adenovirus (AdV), using a single patient sample. Both assays consist of three multiplex real-time PCR (rRT-PCR) panels, and have recently shown good performance in the detection of a battery of respiratory viruses [13–17], but to date they have not been compared to each other.

## Materials and methods

### Assays for virus detection

All NAATs were performed in a manner blinded to the results of the other NAATs.

For Panther Fusion, three different multiplexed rRT-PCR *in vitro* diagnostic tests were used to detect and differentiate respiratory viruses: 1) Fusion Flu A/B/RSV for detection of Flu A, Flu B and RSV; 2) Fusion Paraflu (1/2/3/4) for detection of PIV-1-4; and 3) Fusion AdV/hMPV/RV for detection of AdV, hMPV and RV. Supernatants (500 μl assay volume) were transferred to a specimen lysis tube and loaded directly onto the Panther Fusion System. This platform performs automated nucleic acid extraction and amplification of the gene target sequences by rRT-PCR.

Allplex was composed of three different panels in a multiplex one-step rRT-PCR assay: Panel 1 for detection of Flu A, Flu B, RSV A, RSV B and Flu A subtypes H1, H1pdm09 and H3; Panel 2 for detection of PIV-1, PIV-2, PIV-3, PIV-4, AdV, hMPV and human enterovirus (hEV); and Panel 3 for RV, human coronavirus (hCoV) OC43, 229E and NL63, and human bocavirus (hBoV). Nucleic acid extraction from supernatants (200 μl assay volume) was performed in the Microlab Nimbus IVD (for retrospective samples) or the MicrolabStarlet IVD (for prospective samples) using the STARMag 96 x 4 Universal Cartridge Kit (Seegene, Seoul, South Korea). For rRT-PCR, the CFX96^™^ system (Bio-Rad Laboratories, Hercules, CA, USA) was used. Analysis of the results was done using Seegene viewer software.

The main characteristics of the two tests compared in this study with respect to the degree of automation, test turnaround time, characteristics of the amplification, sample volume, possibility of primary tube utilization, STAT capability and reagent storage conditions are shown in Table 1.

**Table 1:** Main characteristics of Allplex and Panther Fusion systems.

Parameter	Allplex	Panther Fusion
Equipment	Microlab Nimbus/Starlet IVD CFX96 Real-time PCR^1^	Panther Fusion
Automation	For nucleic acid extraction and PCR setup	For nucleic acid extraction, amplification and analysis
Amplification platform	Multiplex one-step RT-PCR	Multiplex one-step RT-PCR
Detection format	Real time MuDT technology	Real time Taqman^™^probes
Hands-on time (min)^2^	45	20
Primary tube utilization	Yes	No
Detection throughput Number of reactions per run	Batches Up to 96	Random Access 60 simultaneous tests
Test turnaround time (h)^3^	4.5	2.5
STAT capability	No	Yes
Sample volume	200 μl	500 μl
Elution volume	100 μl	50 μl
PCR reaction volumen	Final volume: 25 μl Nucleic acid extraction: 8 μl	Final volume^4^: 25–30μl Nucleic acid extraction^4^: 5–10 μl
Number of PCR amplification cycles	45	45
Reagent storage temperature	- 20 ºC	4 ºC

^1^The analysis of the results must be carried out with a specific program (Seegene viewer)

^2^Hands-on time includes time needed for specimen processing

^3^Turnaround time includes time needed for specimen data analysis

^4^For Panther Fusion AdV/hMPV/RV Panel

Both platforms allow visualization of the amplification curves and C_t_ values. The interpretation of the results was carried out in both cases following the manufacturer's instructions.

The instruments used in the assays were provided by the companies, using the standard version that is commercially available.

A third PCR-based assay (R-GENE®, bioMérieux, Marcy l’Etoile, France) [18, 19] using duplex reactions was carried out as a reference method in the case of discordant results. The following R-GENE panels were used: Influenza A/B, RSV/hMPV, Rhino & EV/Cc, AdV/ hBoV and HCoV/HPIV. Nucleic acid extraction from supernatants (200 μl assay volume/50 μl elution volume) was performed using the NucliSENS easyMAG (bioMérieux, Marcy l’Etoile, France). For rRT-PCR, we used the LightCycler 480 System instrument II (Roche Life Science, Indianapolis, IN, USA).

### Study design

The study was divided into two parts, both conducted at the Hospital Universitario 12 de Octubre (Madrid, Spain). A prospective part was conducted during the December 2017-January 2018 flu season, with 405 samples from patients suspected of respiratory viral infection tested simultaneously by both assays. A retrospective part of the study was carried out to test samples for viruses that do not usually circulate during the months of December and January, and also to include viral strains from different years. For this purpose, 400 stored samples were selected in which different respiratory viruses had been detected by more than one diagnostic method in use at the time in our laboratory [19, 20], including rRT-PCR assay and/or shell-vial culture. More than one virus had been previously detected in 39 of these 400 positive samples (9.75%). In addition, the retrospective study included 60 samples in which the presence of common respiratory viruses was ruled out by rRT-PCR.

### Patient characteristics

The samples included in the study belonged to a total of 862 patients (236 pediatric and 626 adult); in 3 patients, 2 samples were tested from different years. 433 (50.2%) patients were male and 429 (49.8%) were female. The median age for the pediatric patients was 1 year (with an interquartile range [IQR] of 0.25–2), whereas for adults it was 71 years (IQR 55–83).

### Specimen collection

A total of 865 clinical specimens were analyzed, of which 563 were nasopharyngeal exudates, 236 nasopharyngeal aspirates (from pediatric patients), 46 BALs and 20 AdV-positive culture supernatants. Nasopharyngeal samples were collected with flocked swabs in UTM^™^ viral transport medium (Copan Diagnostics, Brescia, Italy). For the analysis, samples were vortexed and then swabs were removed from transport devices. Nasopharyngeal aspirates and BAL fluid specimens were diluted 1:1 in UTM viral transport medium upon arrival at the laboratory. All specimens were centrifuged at 3000 xg for 15 min and the supernatant was used for testing.

For the retrospective study, in order to avoid multiple freezing-thawing of the samples, residual supernatants were retrieved from storage at -80 ºC and divided into three aliquots of 500 μl each, and then refrozen at -80 ºC. The first two aliquots of each specimen were thawed to be tested on each instrument, and the third was kept at -80 ºC to be used in the case of discrepancy.

### Data analysis

Samples were considered as true positive (TP) when the viral target was detected in the sample by two of the assays, and true negative (TN) when two of the assays did not detect the viral target. According to these criteria, test results of individual assays were categorized as false positive (FP) or false negative (FN) when the positive or negative result corresponded to a TN or TP sample, respectively. For both primary diagnostic assays, the sensitivity, specificity, positive predictive value (PPV) and negative predictive value (NPV) were calculated. The corresponding two-sided 95% score (Wilson) confidence intervals (CIs) were also estimated, and inter-rater agreement statistics (kappa values; k) were used to compare the detection of the viral targets between the Panther Fusion and Allplex assays.

The median and IQR of C_t_ values from both of the compared assays were calculated for the seven analyzed viruses, and a two-tailed Student’s t test was performed to determine statistical significance. P-values <0.05 were considered to be statistically significant. All calculations were performed on the VassarStats Statistical Computation Web Site (http://vassarstats.net).

### Ethics statement

The study was approved by the Ethical Committee of Hospital Universitario 12 de Octubre (EC number CEIC: 17/379).

## Results

### Retrospective study results

The number of positive samples for each viral agent investigated, is shown in Table 2. In 39/400 samples (9.75%) more than one pathogen was detected. The identification of Flu A subtypes, RSV and PIV types is based on the results obtained with Allplex. For PIV the results were confirmed on Panther Fusion assay.

**Table 2:** Samples included in the retrospective part of the study classified according to their viral etiology and year of collection.

Virus	Number of samples	Respiratory seasons represented (number)	
**Flu A**	**122**	2009–10 2010–11 2011–12 2013–14 2015–16 2016–17	**(n = 6)**
H3N2	61		
H1N1 pdm09	60		
Not subtyped	1		
**Flu B**	**62**	2010–11 2011–12 2012–13 2014–15 2015–16	**(n = 5)**
**RSV**	**64**	2010–11 2011–12 2013–14 2014–15 2015–16 2016–17	**(n = 6)**
RSV A	27		
RSV B	35		
Type not identified	2		
**PIV**	**40**	2009–10 2010–11 2013–14 2014–15 2015–16 2016–17 2017–18	**(n = 7)**
PIV-1	7		
PIV-2	4		
PIV-3	27		
PIV-4	2		
**hMPV**	**43**	2013–14 2014–15 2015–16 2016–17	**(n = 4)**
**AdV** (20 cultured isolates were included	**57**	2009–10 2010–11 2013–14 2014–15 2015–16 2016–17 2017–18	**(n = 7)**
**RV**	**66**	2015–16 2016–17 2017–18	**(n = 3)**
**Negative**	**60**	2015–16 2016–17 2017–18	**(n = 3)**

The TP, TN, FP and FN results obtained by both tests for each of the viruses are shown in Table 3. Globally, both tests showed excellent sensitivity and specificity values; Panther Fusion had sensitivity values >0.95 for all viral targets, whereas Allplex showed values lower than 0.95 only for detection of hMPV and RV, though still >0.90.

**Table 3:** Retrospective comparison of Panther Fusion and Allplex for detection of viruses in stored clinical samples.

Viral Target	TP^1^	TN^2^	Panther Fusion Results				Allplex Results			
			FP^3^	FN^4^	Sensitivity (95% CI)	Specificity (95% CI)	FP^3^	FN^4^	Sensitivity (95%CI)	Specificity (95% CI)
**Flu A**^**5**^	122	338	1	0	1 (0.96–1)	0.99 (0.98–0.99)	0	1	0.99 (0.95–0.99)	1 (0.98–1)
**Flu B**	62	398	0	0	1 (0.92–1)	1 (0.98–1)	0	0	1 (0.92–1)	1 (0.98–1)
**RSV**^**5**^	64	396	2	1	0.98 (0.90–0.99)	0.99 (0.97–0.99)	0	2	0.96 (0.88–0.99)	1 (0.99–1)
**ADV**	57	403	1	0	1 (0.92–1)	0.99 (0.98–0.99)	2	2	0.96 (0.86–0.99)	0.99 (0.98–0.99)
**hMPV**	43	417	7	0	1 (0.89–1)	0.98 (0.96–0.99)	1	3	0.93 (0.81–0.98)	0.99 (0.98–0.99)
**RV**	66	394	3	1	0.98 (0.90–0.99)	0.99 (0.97–0.99)	10	5	0.92 (0.82–0.97)	0.97 (0.95–0.98)
**PIV**^**5**^	40	420	3	0	1 (0.89–1)	0.99 (0.97–0.99)	0	0	1 (0.89–1)	1 (0.98–1)
**TOTAL**	**454**	**2,766**	**17**	**2**	**0.99** (0.97–0.99)	**0.99** (0.98–0.99)	**13**	**13**	**0.97** (0.95–0.98)	**0.99** (0.98–0.99)

^1^TP: true positive

^2^TN: true negative

^3^FP: false positive

^4^FN: false negative

^5^No differences were found between the types of RSV or PIV and the subtypes of Flu A, so the data were analyzed together

**Flu A,** virus influenza A; **Flu B,** virus influenza B; **RSV,** Respiratory Syncytial Virus; **AdV**, Adenovirus; **hMPV**, human metapneumovirus; **RV**, Rhinovirus; **CI**, confidence interval

For 45 samples (9.8%), there was a discrepancy between the two assays. Allplex detected 11 RV positives that were not detected in Panther Fusion, and only one was confirmed by R-GENE. On the other hand, Panther Fusion detected hMPV in 10 samples that were not positive by Allplex, and only 3 of them were confirmed by R-GENE. Regarding the negative control samples in which no respiratory viruses had been previously identified, Allplex did not detect any viral agent, while Panther Fusion had a false positive result when detecting an AdV that was not confirmed by R-GENE.

### Prospective study results

Six invalid samples were detected in Allplex Panel 1 and one sample in Allplex Panels 2 and 3 (n = 7, 1.73%), while a total of five samples were invalid in the Panther Fusion AdV/hMPV/RV and/or Paraflu (1/2/3/4) panels (n = 5, 1.23%). After discarding all samples with any invalid results, a total of 399 samples were analyzed for Flu A and B, RSV, AdV, hMPV and RV, and 402 samples for PIV. In 20 samples (4.9%), more than 1 virus was detected.

The overall results obtained by both primary assays for each virus are shown in Table 4.

**Table 4:** Comparison of Panther Fusion and Allplex for detection of viruses in patient samples collected from December 2017 to January 2018.

Viral Target	TP^1^	TN^2^	Panther Fusion Results						Allplex Results					
			FP^3^	FN^4^	Sensitivity (95% CI)	Specificity (95% CI)	PPV^5^ (95% CI)	NPV^6^ (95% CI)	FP^3^	FN^4^	Sensitivity (95% CI)	Specificity (95% CI)	PPV^5^ (95% CI)	NPV^6^ (95% CI)
**Flu A**	72	327	7	0	1 (0.93–1)	0.97 (0.95–0.99)	0.91 (0.82–0.96)	1 (0.98–1)	7	5	0.93 (0.83–0.97)	0.97 (0.95–0.99)	0.90 (0.80–0.95)	0.98 (0.96–0.99)
**Flu B**	70	329	5	2	0.97 (0.89–0.99)	0.98 (0.96–0.99)	0.93 (0.84–0.97)	0.99 (0.97–0.99)	1	4	0.94 (0.85–0.98)	0.99 (0.98–0.99)	0.98 (0.91–0.99)	0.98 (0.96–0.99)
**RSV**	38	361	0	0	1 (0.88–1)	1 (0.98–1)	1 (0.88–1)	1 (0.98–1)	0	4	0.89 (0.74–0.96)	0.91 (0.88–0.93)	1 (0.87–1)	0.98 (0.97–0.99)
**ADV**	8	391	1	0	1 (0.59–1)	0.99 (0.98–0.99)	0.88 (0.50–0.99)	1 (0.98–1)	3	0	1 (0.59–1)	0.99 (0.97–0.99)	0.72 (0.39–0.92)	1 (0.98–1)
**hMPV**	6	393	3	1	0.83 (0.36–0.99)	0.99 (0.97–0.99)	0.62 (0.25–0.89)	0.99 (0.98–0.99)	1	1	0.83 (0.36–0.99)	0.99 (0.98–0.99)	0.83 (0.36–0.99)	0.99 (0.98–0.99)
**RV**	37	362	5	2	0.94 (0.80–0.99)	0.98 (0.96–0.99)	0.87 (0.72–0.95)	0.99 (0.97–0.99)	13	2	0.94 (0.80–0.99)	0.96 (0.93–0.97)	0.72 (0.57–0.84)	0.99 (0.97–0.99)
**PIV**	13	389	3	0	1 (0.74–1)	0.99 (0.97–0.99)	0.83 (0.57–0.95)	1 (0.98–1)	4	1	0.93 (0.66–0.99)	0.98 (0.97–0.99)	0.77 (0.51–0.92)	0.99 (0.98–0.99)
**TOTAL**	**244**	**2,552**	**24**	**5**	**0.97** (0.95–0.99)	**0.98** (0.98–0.99)	**0.91** (0.86–0.94)	**0.99** (0.99–0.99)	**29**	**17**	**0.93** (0.89–0.95)	**0.98** (0.98–0.99)	**0.88** (0.84–0.92)	**0.99** (0.98–0.99)

^1^TP: true positive

^2^TN: true negative

^3^FP: false positive

^4^FN: false negative

^5^PPV: positive predictive value

^6^NPV: negative predictive value.

**Flu A,** virus influenza A; **Flu B,** virus influenza B; **RSV,** Respiratory Syncytial Virus; **AdV**, Adenovirus; **hMPV**, human metapneumovirus; **RV**, Rhinovirus; **CI**, confidence interval

The two primary assays showed negative results for all the viruses investigated in 150 samples (37.0%), and discrepant results in 75 samples (18.8%). Regarding sensitivity and specificity, both methodologies yielded high values, ranging from 0.83–1 with good predictive values, although, in general, Panther Fusion tended to be more sensitive. Comparing the viral targets included in the Flu A/B/RSV panels, the values reached by Panther Fusion for the detection of RSV stand out. Both assays presented similar values for hMPV and RV, coinciding with the findings of the retrospective study.

The Allplex assay allowed for some differentiation of the results based on subtype. Of the 67 samples positive for Flu A, 41 were H1pdm09, 28 were H3, and 1 wasn’t subtyped. For RSV, 8 were RSV A and 26 were RSV B. In 26 samples (6.5%), Allplex detected other viruses that were not included in the diagnostic capability of Panther Fusion: hCoV OC43 (n = 18), hCoV NL63 (n = 8), hEV (n = 2) and hBoV (n = 13).

### Agreement between the Allplex and Panther Fusion assays

Specific comparisons of both methods for the seven viruses were performed, analyzing the results obtained in both the prospective and retrospective parts of the study. Overall agreement ranged from 97.55–98.9%, with the lowest k values determined for hMPV (k = 0.83, 95% CI: 0.75–0.91) and RV (k = 0.73, 95% CI: 0.65–0.81) (Table 5).

**Table 5:** Performance of Allplex and Panther Fusion systems for each viral target analyzed in both retrospective and prospective studies.

	Panther Fusion				
Allplex	Positive *n* (%)	Negative *n* (%)	Total *n* (%)	Agreement (%)	Cohen´s k (95% CI)
**Flu A**				**97.55**	**0.93 (0.90–0.96)**
**Positive, *n* (%)**	187 (21.8)	7 (0.8)	194 (22.7)		
**Negative, *n* (%)**	14 (1.6)	651 (75.8)	665 (77.3)		
**Total, *n* (%)**	201 (23.4)	658 (76.6)	859 (100.0)		
**Flu B**				**98.6**	**0.95 (0.92–0.98)**
**Positive, *n* (%)**	128 (14.9)	3 (0.4)	131 (15.3)		
**Negative, *n* (%)**	9 (1.0)	719 (83.7)	728 (84.7)		
**Total, *n* (%)**	137 (16.0)	721 (84.0)	859 (100.0)		
**RSV**				**98.9**	**0.95 (0.91–0.98)**
**Positive, *n* (%)**	94 (11.0)	1 (0.1)	95 (11.1)		
**Negative, *n* (%)**	8 (0.9)	756 (88.0)	764 (88.9)		
**Total, *n* (%)**	102 (11.9)	757 (88.1)	859 (100.0)		
**AdV**				**98.9**	**0.92 (0.87–0.97)**
**Positive, *n* (%)**	58 (6.7)	5 (0.6)	63 (7.3)		
**Negative, *n* (%)**	4 (0.5)	792 (92.2)	796 (92.7)		
**Total, *n* (%)**	62 (7.2)	797 (92.8)	859 (100.0)		
**hMPV**				**98.0**	**0.83 (0.75–0.91)**
**Positive, *n* (%)**	44 (5.2)	3 (0.3)	47 (5.5)		
**Negative, *n* (%)**	14 (1.6)	798 (92.9)	812 (94.5)		
**Total, *n* (%)**	58 (6.8)	801 (93.2)	859 (100.0)		
**RV**				**98.7**	**0.73 (0.65–0.81)**
**Positive, *n* (%)**	63 (7.4)	26 (3.0)	89 (10.4)		
**Negative, *n* (%)**	15 (1.7)	755 (87.9)	770 (89.6)		
**Total, *n* (%)**	78 (9.1)	781(90.9)	859 (100.0)		
**PIV**				**98.6**	**0.92 (0.84–0.96)**
**Positive, *n* (%)**	52 (6.0)	4 (0.5)	56 (6.5)		
**Negative, *n* (%)**	7 (0.8)	799 (92.7)	806 (93.5)		
**Total, *n* (%)**	59 (6.8)	803 (93.2)	862 (100.0)		

**Flu A,** virus influenza A; **Flu B,** virus influenza B; **RSV,** Respiratory Syncytial Virus; **AdV**, Adenovirus; **hMPV**, human metapneumovirus; **RV**, Rhinovirus; **CI**, confidence interval

### Amplification efficiency

As both methods require the same number of amplification cycles, amplification efficiency was then evaluated by comparing the median C_t_ values between assays (Table 6). For the comparison of the results obtained for the amplification of Flu A, in the case of Allplex the C_t_ obtained in the generic amplification of the virus was used. For four of the seven viruses analyzed (Flu A, Flu B, hMPV and RV), Panther Fusion showed significantly lower median C_t_ values compared to Allplex (*p* <0.001)

**Table 6:** Comparison of amplification efficiency for Allplex and Panther Fusion systems.

Viral target	Allplex Median C_t_ value (IQR)	Panther Fusion Median C_t_ value (IQR)	*p* value
Flu A^1^	30.28 (26.91–34.31)	24.50 (21.20–28.90)	**< 0.001**
Flu B	32.13 (28.90–36.85)	27.50 (24.60–34.70)	**< 0.001**
RSV	27.95 (23.87–34.77)	27.10 (22.70–30.50)	0.21
AdV	18.65 (15.73–25.49)	17.70 (14.50–24.70)	0.17
hMPV	32.61 (30.36–35.79)	26.45 (23.85–30.65)	**< 0.001**
RV	33.13 (27.65–38.03)	28.10 (21.20–33.0)	**< 0.001**
PIV	27.79 (23.16–33.17)	28.80 (23.05–34.45)	0.39

^1^ Median value calculated using the generic amplification values of influenza A virus

**Flu A,** virus influenza A; **Flu B,** virus influenza B; **RSV,** Respiratory Syncytial Virus; **AdV**, Adenovirus; **hMPV**, human metapneumovirus; **RV**, Rhinovirus; **IQR**, interquartile range

### Discordant results

Finally, discordant results were interpreted by considering the results of the first two assays along with those of the R-GENE assay (Table 7). Discordant results for all viruses analyzed were found, being more frequent when RV (n = 41 samples), Flu A (n = 21), and hMPV (n = 17) were tested. Regarding the Flu A virus, the discordance for the Allplex assay was not associated with any subtype or with the detection of non-typeable strains.

**Table 7:** Discordant results between Allplex and Panther Fusion and results obtained with the reference method (R-GENE).

Viral target	Allplex (+) / Panther Fusion (-)	Confirmed by R-GENE	Allplex (-) / Panther Fusion (+)	Confirmed by R-GENE	*p*-value
**Flu A**	7	0	14	6 (42.9%)	**0.04**
**Flu B**	3	2 (66.7%)	9	4 (44%)	0.5
**RSV**	1	1 (100%)	8	6 (75%)	0.57
**AdV**	5	0	4	2 (50%)	0.07
**hMPV**	3	1 (33.3%)	14	4 (28.6%)	0.87
**RV**	26	3 (11.5%)	15	5 (33.3%)	0.089
**PIV**	4	0	7	1 (14.3%)	0.43
**Total**	**49**	**7 (14.3%)**	**71**	**28 (39.4%)**	**0.11**

**Flu A,** virus influenza A; **Flu B,** virus influenza B; **RSV,** Respiratory Syncytial Virus; **AdV**, Adenovirus; **hMPV**, human metapneumovirus; **RV**, Rhinovirus.

**Fig 1:**
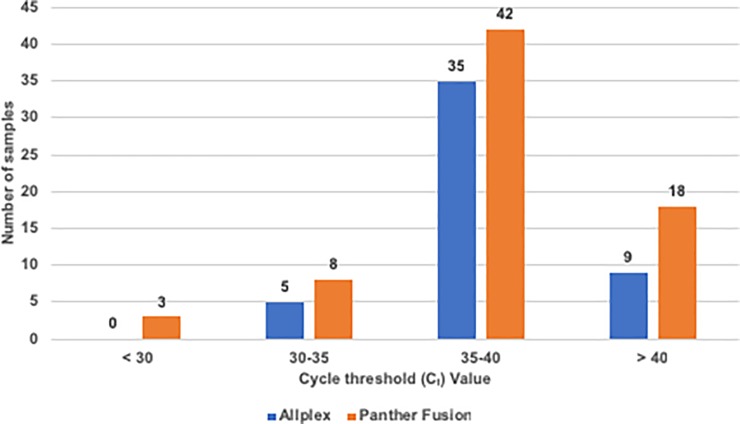
Distribution of samples with discordant results according to their cycle threshold (C_t_) values.

The C_t_ value obtained in both assays was analyzed in the 120 samples with discordant results (Fig 1); 117 samples presented values of C_t_ >30 in both tests, with most of them (35/49 [71.4%] for Allplex and 42/71 [59.1%] for Panther Fusion) showing a C_t_ value between 35 and 40 cycles, whereas only 3 samples with a C_t_ <30 in Panther Fusion were discordant. These results indicate that samples with discordant results had a low viral load, as expected.

Overall, the reference method showed higher agreement with Panther Fusion-positive specimens (negative for Allplex; *n* = 28, 39.4%) than with Allplex-positive samples (negative for Panther Fusion; *n* = 7, 14.3%), although this difference was only statistically significant for Flu A (*p* = 0.04).

## Discussion

Respiratory viral infections are a major global health problem today [21], and they require accurate diagnostic tools which must be thoroughly evaluated prior to their implementation in routine laboratory practice. In this study, we compared the performance of the Panther Fusion assays with Allplex respiratory panels (approved by Conformité Européenne-*in vitro* diagnostics [CE-IVD]), using R-GENE technology as a reference method.

Our results demonstrate that both technologies produced comparable results for the detection of seven viruses which are responsible for the majority of viral respiratory infections [22–24], with the Panther Fusion respiratory assays showing slightly better sensitivity than the Allplex panels. Furthermore, Panther Fusion showed better amplification efficiency for Flu A and B, hMPV and RV detection, which may be explained by differences in the detection technology used or differences in targeted genes. Although the overall agreement between both methods was high, lower values were observed for RV and hMPV, two viruses with great genomic diversity [25–26]. It is noteworthy that the discordant results detected with both methods were associated with high C_t_ values, suggesting a low viral genomic load in the clinical samples, close to the limit of detection of the assays.

Several multiplexed respiratory assays with CE-IVD approval are currently available on the market. Among them, Allplex respiratory assays have shown good performance and reliable results [15–17]. The recently-developed Panther Fusion System is a fully automated random-access testing system that has shown comparable results with those obtained with the eSensor RVP (eSensor; Genmark Dx, Carlsbad, CA) and Lyra (Quidel, San Diego, CA) respiratory assays [13]. Our results show that this is also true when compared to Allplex technology, a method that can detect a broader variety of viruses in a single channel, but requires more hands-on time and consequently slower turnaround time. The Panther Fusion assays showed greater agreement with the third, reference rRT-PCR method than the Allplex panels, which indicates slightly better specificity for the targeted viruses.

In this study, every type of clinical respiratory sample (nasopharyngeal aspirates, nasopharyngeal exudates and BALs) was analyzed, resulting in a significant number of samples in which multiple viruses were co-detected by both assays. Furthermore, the methodology used in the retrospective study ensured that, in all tests carried out (including the reference test), the samples were treated under the same conditions, avoiding possible interference caused by repeated freeze-thawing of the samples [13].

In conclusion, Panther Fusion was validated alongside Allplex as a sensitive and specific assay for detecting the most common viruses responsible for respiratory infections, allowing a fully automated rRT-PCR process and random access with a clinically appropriate turnaround time, and thus is a suitable method for implementation in routine clinical viral diagnostics.
